# Aspiration pneumonia in nursing literature—a mapping review

**DOI:** 10.3389/fresc.2024.1393368

**Published:** 2024-07-24

**Authors:** Dominika Lisiecka, Áine Kearns, William Evans, Dawn Farrell

**Affiliations:** ^1^ Department of Nursing and Healthcare Sciences, Munster Technological University—Kerry Campus, Tralee, Ireland; ^2^ Kerry Speech & Language Therapy Clinic, Tralee, Ireland; ^3^ Health Research Institute, School of Allied Health, University of Limerick, Limerick, Ireland

**Keywords:** aspiration pneumonia, nursing, dysphagia, oral hygiene, tube feeding, mapping review

## Abstract

**Introduction:**

Aspiration pneumonia (AP) is an infection of the lungs caused by inhalation of material. The reported incidences vary across literature and clinical populations and is associated with high morbidity and mortality. Management of AP is best carried out by a multidisciplinary team.

**Methods:**

This aim of this review was to collate and describe the available evidence on AP to develop a greater understanding of the concept of AP as it is represented in the nursing literature. As a collaborative team, we undertook the six stages of a systematic mapping review. We searched for the term aspiration pneumonia in 200 peer reviewed nursing journals across 10 databases, over a ten-year period (2013–2023).

**Results:**

In this review, 293 papers were coded. Dysphagia, oral health and tube feeding emerged as the most frequent risk factors for AP, and the most reported factors for preventing this condition. Mortality was the most commonly described consequence of AP, followed by hospitalisations and morbidity. Multiple management approaches were reported including dysphagia assessment, risk evaluation, oral care and texture modification of food and fluids. The role of nurses and interprofessional collaborations were described.

**Discussion:**

Despite limited evidence related to the topic of AP in the nursing literature, the complexity of the causes, prevention, management and consequences of AP emerged. Certain factors, such as dysphagia, oral health, and tube feeding, were described under prevention, cause and management of AP. The importance of multidisciplinary approach in the management and prevention of AP was presented.

## 1 Introduction

Aspiration pneumonia (AP) is a bacterial infection of the lungs caused by the aspiration of pathogens into lungs (1, 2). The examples of fluid which may contain pathogens are oropharyngeal secretions (3). Respiratory symptoms of AP can include tachypnea, dyspnea, cough, adventitious breath sounds, and hypoxia (4). Non-respiratory symptoms, more commonly seen in older adults, include mental status changes, falls, loss of appetite, and altered functional status (4).

AP is not an easy condition to diagnose (5), with absence of a clear definition and clinical consensus (6). The prevalence of AP is difficult to measure due to a lack of biomarkers, therefore the true incidence rate remain unknown (3). A recent paper explained challenges associated with distinguishing AP from hospital acquired pneumonia and community acquired pneumonia (6). The authors proposed to call pneumonia occurring in older frail people frailty-associated pneumonia (6).

AP is most prevalent in people with a learning disability, neurological or upper gastrointestinal conditions, and older adults (5). Conditions that increase the risk for AP include stroke, drug overdose, alcohol abuse, seizures, general anaesthesia, head trauma, intracranial masses, dementia, oesophageal strictures, gastroesophageal reflux, pseudobulbar palsy, tracheostomy, NG tube feeding, bronchoscopy and protracted vomiting (3). The evidence highlights the possible association between poor oral health and a risk of AP (7, 8), especially in older adults (8, 9). Oral health is essential for the overall health and wellbeing of individuals and particularly older adults who often reside in care settings and rely on caregivers to support oral health practices (9). Furthermore, this vulnerable group often have more limited access to professional oral health care (9). The evidence also confirms a positive relationship between the presence of AP with dysphagia (5, 6, 8, 10). Bosch et al. (2022) found that dysphagia was most frequently associated with the diagnosis of AP (48.2%), with dysphagia significantly linked to AP in hospitalised patients (11).

The consequences of AP include morbidity and mortality as well as a prolonged hospital stay (3, 5, 12) and increased treatment cost (13, 14). The outcomes depend on the volume of aspiration, patient age, general lung health, presence of any comorbidity, and time to diagnosis (15). The management and prevention of AP require a collaborative multidisciplinary team approach and nurses and speech & language therapists are important members of this team (3, 5, 16, 17). It is known that increasing the knowledge of nursing staff regarding evidence-based care for the prevention and management of AP, particularly in groups at risk of dysphagia, will improve patient outcomes and reduce the incidence of AP and mortality (18). Yet, there is little insight into the focus of nursing literature on the topic of AP. Therefore, we were interested to investigate how AP is represented in the nursing literature.

## 2 Methods

The aim of this review was not to ask or answer a specific research question on AP. Instead, the aim was to collate and describe available evidence to develop a greater understanding of the concept of AP as it is represented in the nursing literature. Therefore, a mapping review methodology was considered appropriate for this review due to the anticipated heterogeneity of the available research within the field of nursing. The mapping review method was developed by the Evidence for Policy and Practice Information and Co-ordinating Centre (EPPI-Centre), Institute of Education, London, to investigate research on a broad subject of interest (19, 20). This type of review aims to categorise, describe and map available evidence on a broad subject of interest, into an inductively developed framework. There is no standardised guidance document available equivalent to the PRISMA-P for mapping review protocols. Therefore, the methodology used in this review aligned with many of the processes described in James et al. (19) and O'Cathain et al. (21).

### 2.1 Establishing the review team and the search strategy

James et al. describes six stages of systematic mapping processes (19). Stage 1 of the review process involves the establishment of the team. In this review, the team included researchers with experience in a range of review methodologies and with professional backgrounds in nursing and speech and language therapy. In this first stage, the scope of the review was discussed and defined. The scope was intended to cover a broad review that explored the term aspiration pneumonia in the nursing literature over a ten-year period (2013–2023). When searching the evidence, as part of Stage 2, the authors obtained the list of all nursing journals available through the library of a higher education institution in the Republic of Ireland that provides pre- and post-registration nursing education programmes. During this stage, 453 journals were identified. Of these, 200 journals were categorised as peer-reviewed. During October 2023, the term aspiration pneumonia was searched within each of these journals.

### 2.2 Screening

Stage 3 involves screening and full text retrieval. However, due to the nature of the research question which sought to map the available evidence on AP in nursing literature, the title and abstracts were not screened. Instead, all retrieved records were added to EndNote and full texts were retrieved, where possible. A small number of full texts could not be retrieved and these records were excluded from the analysis. In addition, records of conference proceedings, book chapters, and duplicates were also removed.

### 2.3 Coding

In order to progress to Stage 4, which involves coding and production of an evidence map, the records were imported into NVivo. The data extracted from the articles included: country, year of publication, context of the study (e.g., population, setting etc.). In addition, three key pieces of data were extracted and analysed: frequency of term aspiration pneumonia, location of term within the article and additional information regarding AP. The latter was coded using a coding framework developed by the research team. It included codes related to cause and risk factors, prevention, management and consequences of AP. If papers clearly identified the knowledge gaps and recommended future research directions in relation to AP, these data were also extracted. A sample of 55 papers (10%) were independently coded by three researchers in order to develop the coding framework. Any disagreements were discussed and resolved through consensus. The remaining texts were coded first by one researcher using the agreed framework to produce a systematic map database within NVivo. A second researcher then coded the content under subcategories of codes within the coding framework.

### 2.4 Describing the findings

Stage 5 is an optional critical appraisal stage as quality assessment of included papers is not required in mapping reviews (19, 20). Due to the nature of the review question and the heterogeneity among research designs within the included articles of the review, this stage was not considered appropriate for this review. The final stage, Stage 6, requires the research team to describe the findings. Here, the systematic map database can be used to describe the scope of the research and identify knowledge clusters and gaps (19). Descriptive statistics, tables and charts were utilised as helpful ways of easily visualising the data. An online data mapping software programme was used to produce a choropleth map of countries included in the review (https://www.datawrapper.de/maps/choropleth-map).

## 3 Results

**Figure 1:**
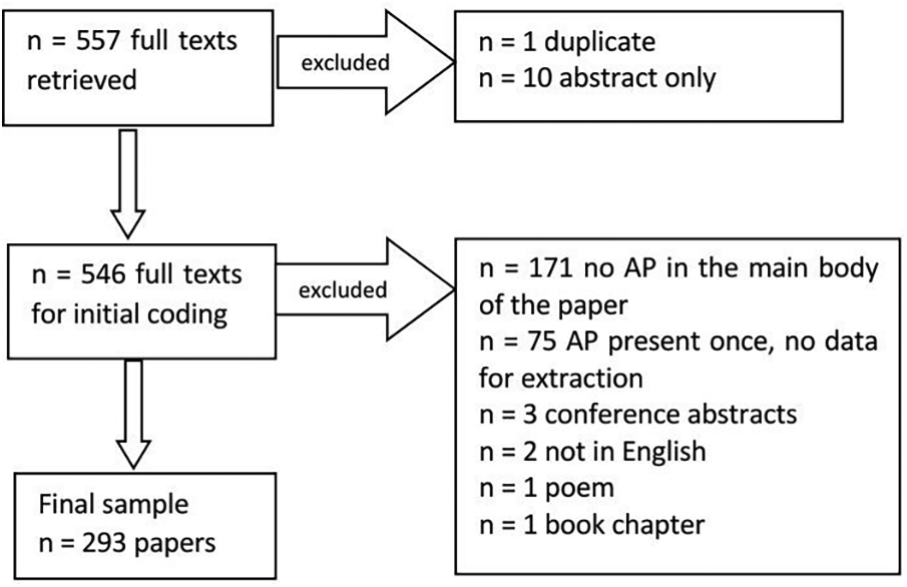
Data screening flowchart.

We identified 200 peer reviewed nursing journals across 10 databases in the University's library. We searched for the term aspiration pneumonia within each journal, which identified 605 records (for details see Supplementary Appendix S1). We were able to obtain a full text for 557/605 records. Papers were excluded for the following reasons: abstracts only (*n* = 10), duplicates (*n* = 1). During the coding phase, an additional 253 records were excluded due to the following reasons: no AP in the body of the paper (*n* = 171), AP reported once in the paper without sufficient information which would enable the coding (*n* = 75), conference abstract (*n* = 3), Chinese language (*n* = 2), a poem (*n* = 1), a book chapter (*n* = 1). The final sample consisted of *n* = 293 (see Figure 1). The data are presented below in the format based on the coding framework developed by the authors' team. The most prevalent themes are described in text and supplemented by figures.

### 3.1 The overall characteristic of the sample

There was a high heterogeneity of papers, including experts' opinion articles, primary and secondary research papers, practice guidelines and recommendations. In 55% of records (*n* = 162) the term AP was reported once only within the body of the paper. However, there was sufficient information provided to allow for content coding. In terms of the location of the term aspiration pneumonia within the papers (i.e., whether this term was present in the introduction and background, methods, results or discussion sections), we were able to establish this for 167 (57%) of the sample (the remaining *n* = 126 papers did not use the IMRaD structure). In the 167 records, the term aspiration pneumonia was reported 204 times in the background and introduction to the paper, 29 times in the methods section, 74 times in the results section, and 93 times in the discussion.

**Figure 2:**
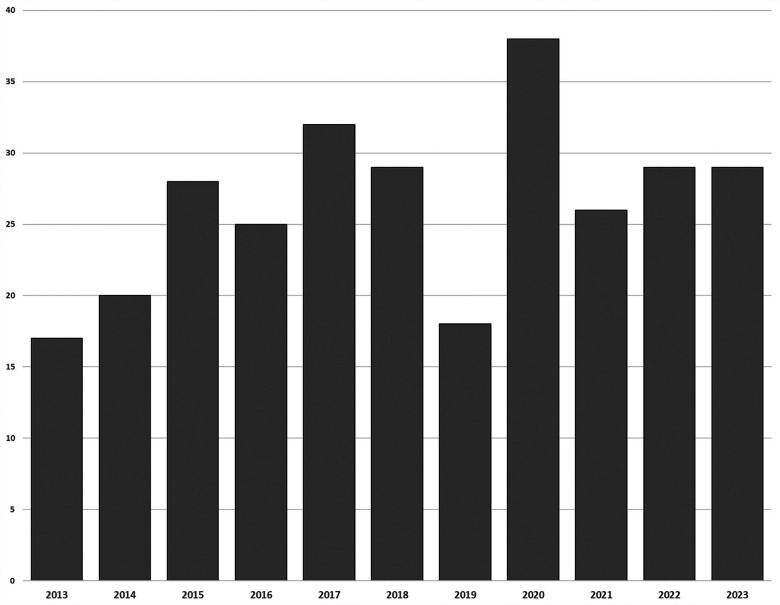
Frequency of publication.

Within the included papers, the average number of publications per year is 26. This drops to a low of 17 in 2013, and 18 in 2019, while the highest publication rate is 2020 when there were 38 papers (Figure 2).

### 3.2 Countries

**Figure 3:**
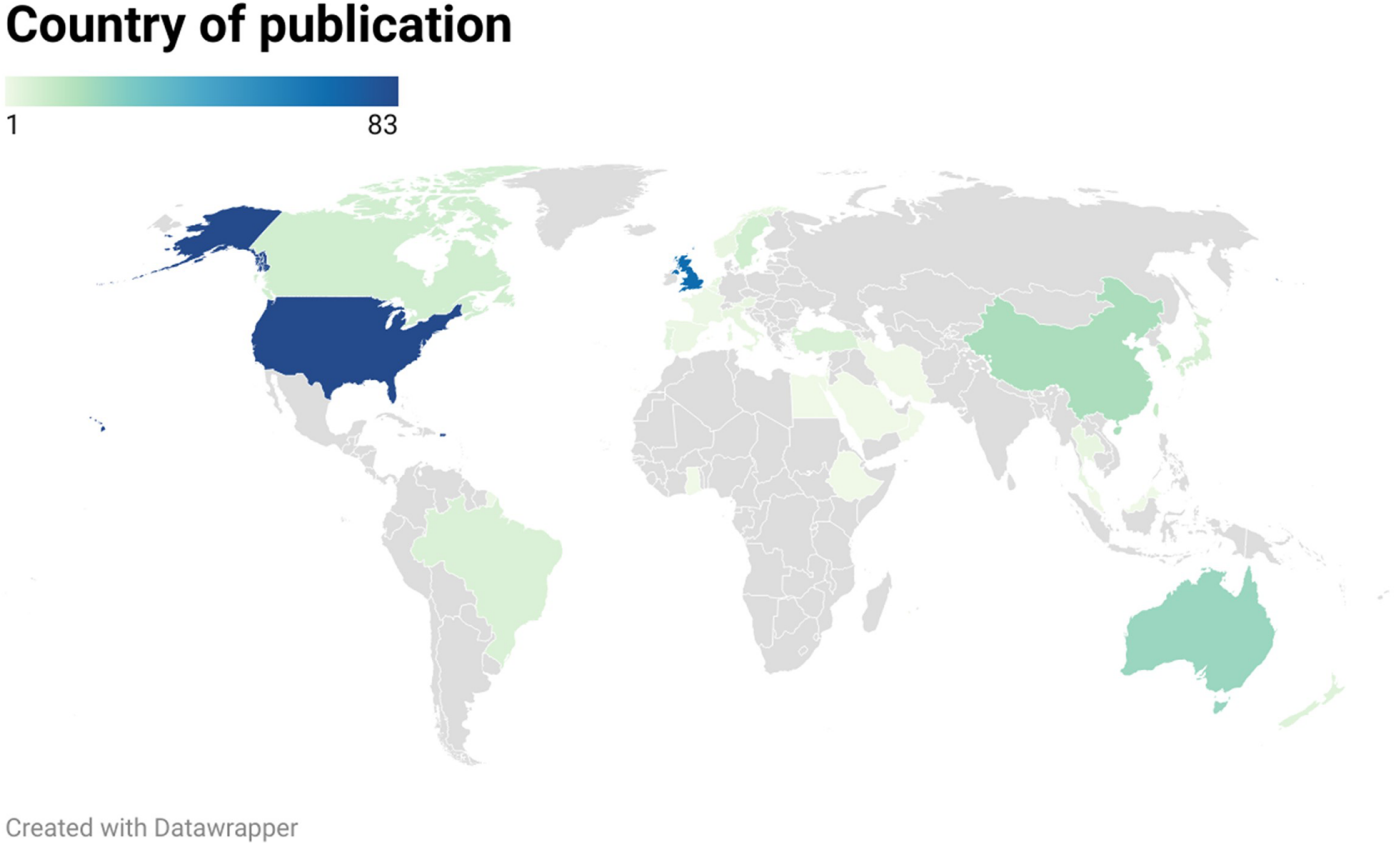
Location of papers.

In terms of the geographical spread of the papers included in this review, there was representation of all continents within the included papers. The majority came from United States of America (*n* = 83, 28%), followed by the United Kingdom (*n* = 68, 23%), Australia (*n* = 21, 7%), China (*n* = 17, 6%), Korea (*n* = 12, 4%), and Taiwan (*n* = 10, 3%) (Figure 3).

### 3.3 Context

**Figure 4:**
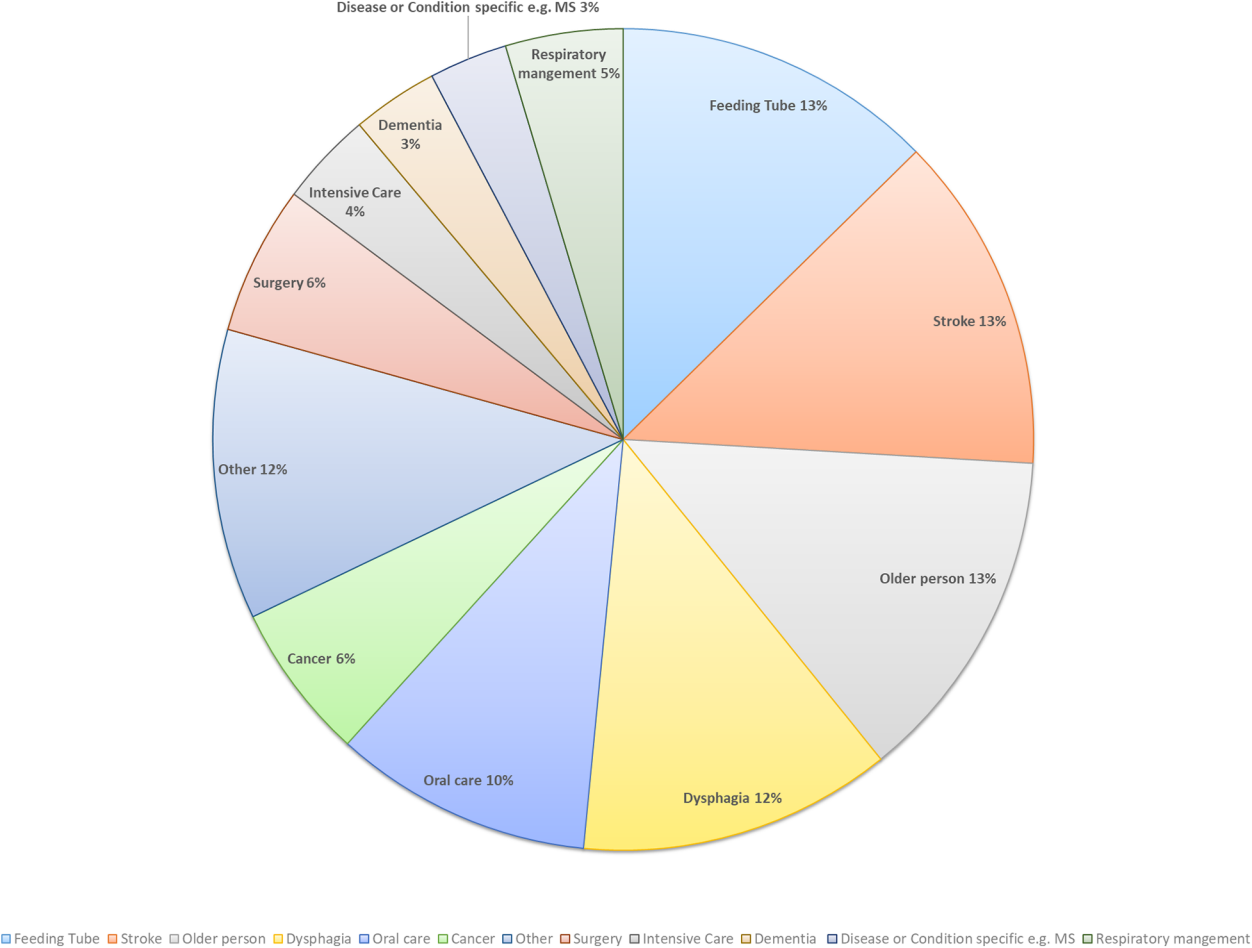
Context of papers.

Due to the high heterogeneity of papers, the context, setting, and populations could not be easily identified for all records. We noted a higher prevalence of papers on the topic of tube feeding, stroke, older person, dysphagia, and oral hygiene (Figure 4). The included papers focused on the range of populations across the lifespan, but were primarily focused on adult. The vast majority of papers were not focused on AP. There were twenty studies that included the term aspiration pneumonia in their methods section (this term occurred 29 times across the twenty papers, as per Section 3.1). The term was searched within a chart or medical records review (14, 22–24), as well as in a literature review (25, 26). Two studies referenced AP in a case description (22, 27). AP was listed as an outcome when investigating oral care (28, 29) and gastric residual volume (23, 30). AP was listed as a diagnosis for some participants in two studies (31, 32). It was also listed as a secondary outcome when investigating gastrointestinal ulcers (33). One study investigated reasons for developing AP and constipation (34). AP was also listed as a symptom when investigating the prevalence of dysphagia (35), included to develop clinical practice guidelines for aspiration (36), and to translate and validate the Self-Care for Aspiration Pneumonia Prevention Scale (37). AP was listed as an exclusion criterion in two studies (38, 39).

### 3.4 The cause and risk factors of AP

**Figure 5:**
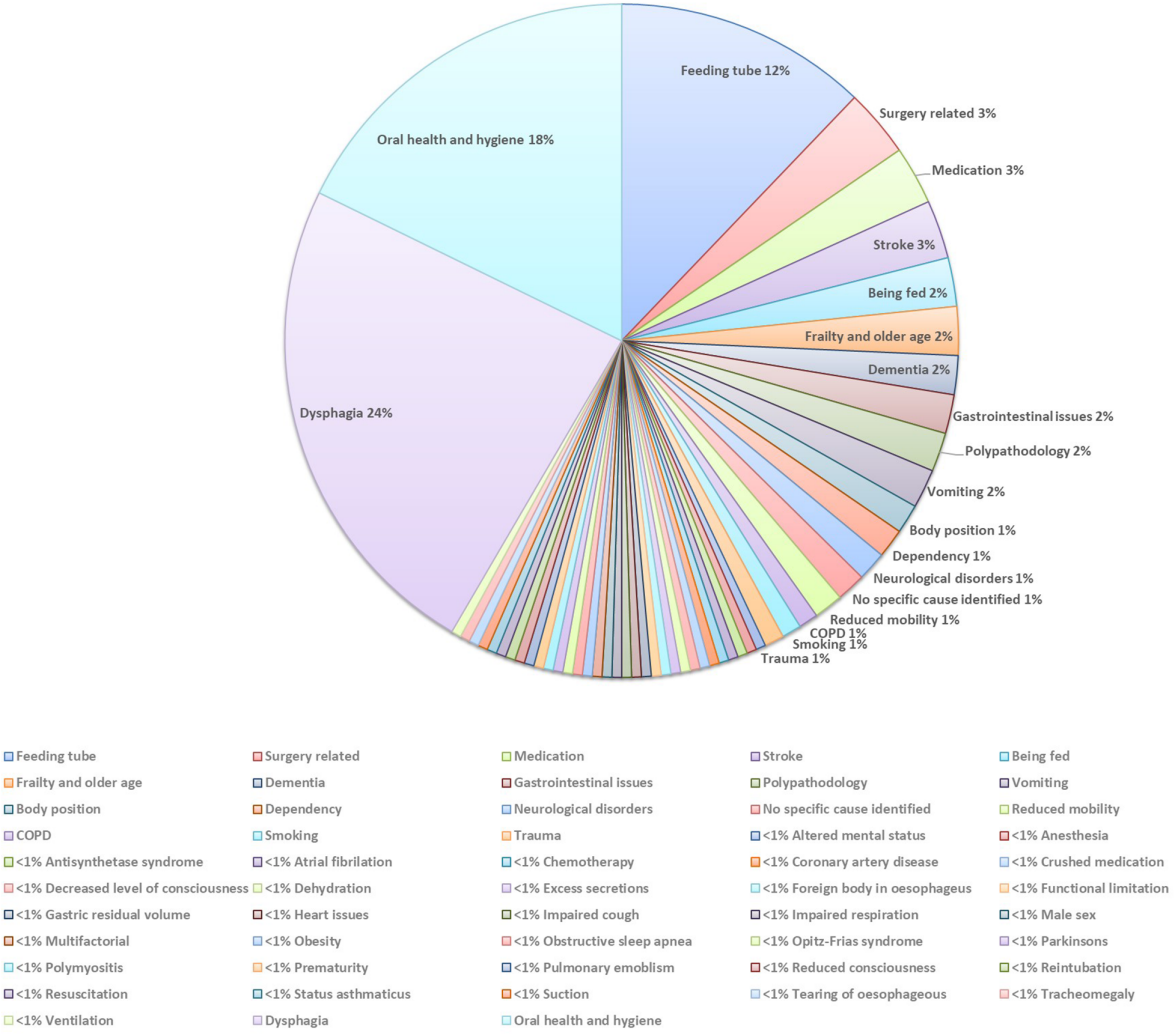
The cause and risk factors of AP.

Over half (52%) of the papers reported the cause and risk factors for developing AP. The top three were dysphagia, poor oral health and hygiene, and tube feeding. The most prevalent cause was dysphagia, represented in 51 papers (2, 8, 14, 17, 31, 33, 35, 40–83). This was followed by poor oral health and hygiene, reported in 38 papers (1, 2, 17, 28, 29, 45, 48, 51, 57, 59, 66, 68, 82–108), and tube feeding, noted in 27 papers (2, 12, 17, 26, 51, 60, 63, 72, 109–126). A number of papers (*n* = 33) presented single occurrence causes, for example obstructive sleep apnoea (127) or male gender (51) (Figure 5).

### 3.5 The management of AP

**Figure 6:**
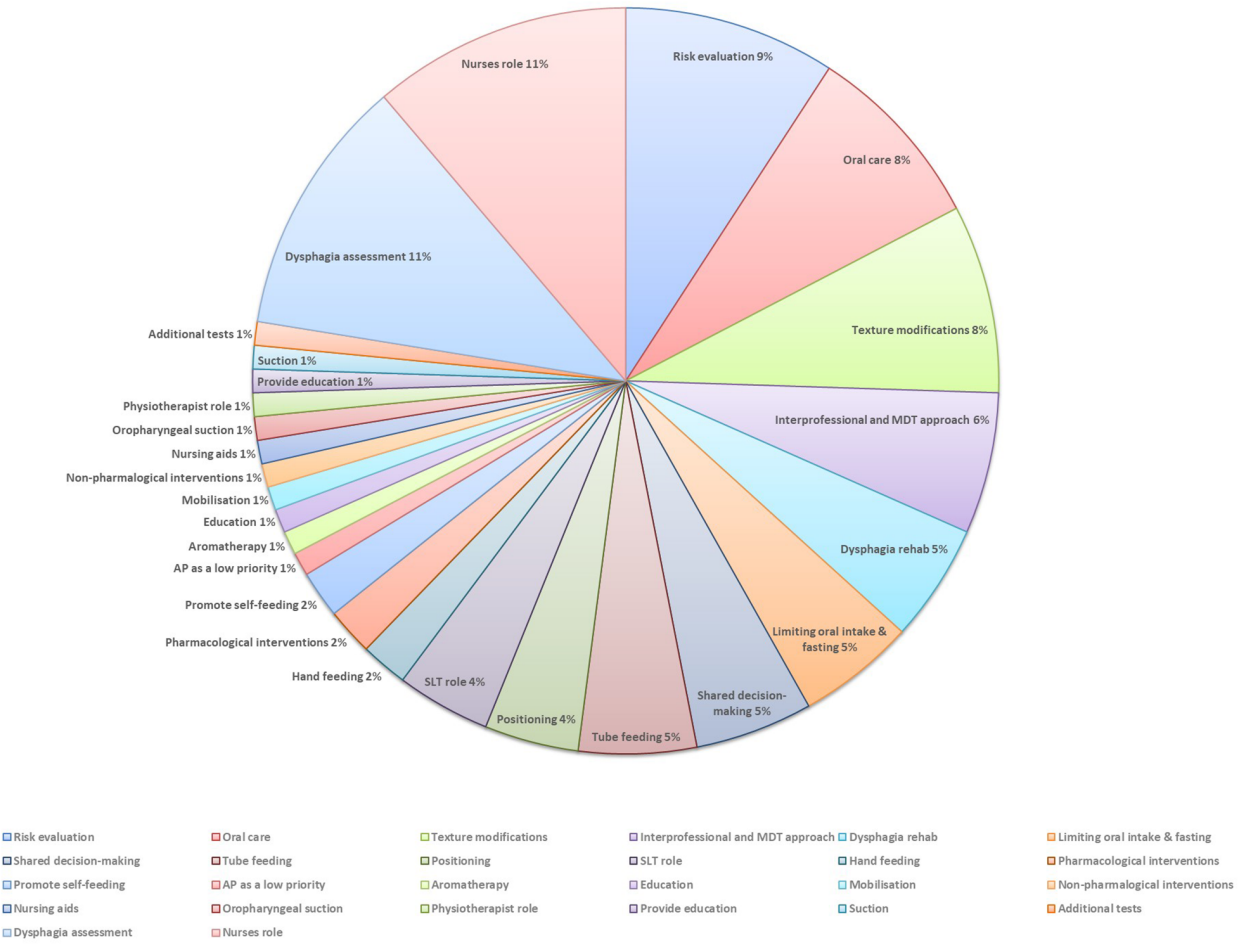
The management of AP.

Fifty-two papers (18%) reported on the management of people with AP (Figure 6). Within these papers, there were descriptions of the management of people with AP co-occurring with accounts of the prevention of this condition. The papers described a multidisciplinary approach when managing people with or at risk of AP. The team members included nurses (4, 14, 35, 42, 57, 95, 113, 128–132), speech & language therapists (8, 57, 88, 132–134), physiotherapist (34), and nursing aids (135). Nurses were identified as holding many responsibilities, for example, to identify and monitor the changes in a patient's status (14, 57, 131), coordinate input from other professionals (14, 57), make appropriate multidisciplinary referrals (14). Nurses were also responsible for providing education on the management of AP (129), assessing or screening for dysphagia (113, 132), helping to balance quality of life with medical risk associated with AP (35), providing oral care (4, 128), correct positioning (4), and support at mealtimes (4, 42).

Dysphagia assessment was presented as important in managing people with or at risk of AP. Through the identification of people with swallowing problems, dysphagia assessment could help to reduce the negative impact of AP (49, 136, 137), such as morbidity and mortality (49, 137). Food and fluid texture modifications were noted in the management of people with dysphagia who have or are at risk of AP (57, 131, 138–143). However, their benefits were reported as questionable (138), with one paper stating that thickened fluid “does not reduce AP in all patients and can pose other risks for physical health and psychological well-being” (57).

Risk mitigation or evaluation in the context of the management of AP emerged in some papers (8, 35, 49, 138, 144–148), and the importance of shared decision-making and balancing quality of life with a medical risk of AP was outlined (35, 138, 146). Risk of AP was predominantly linked with the presence of dysphagia (8, 35, 138, 145, 146, 148).

Tube feeding was also noted within the papers that referenced managing people with AP. While some papers stated that tube feeding, such as nasogastric tube, is introduced due to AP (14, 113), others reported that this does not reduced the risk of AP (144, 149).

The importance of adequate oral care for people with or at risk of AP was reported (139, 98, 92, 150, 39, 90, 151, 152). The included papers identified the implementation of oral hygiene in both the management and prevention of AP.

### 3.6 The consequences of AP

**Figure 7:**
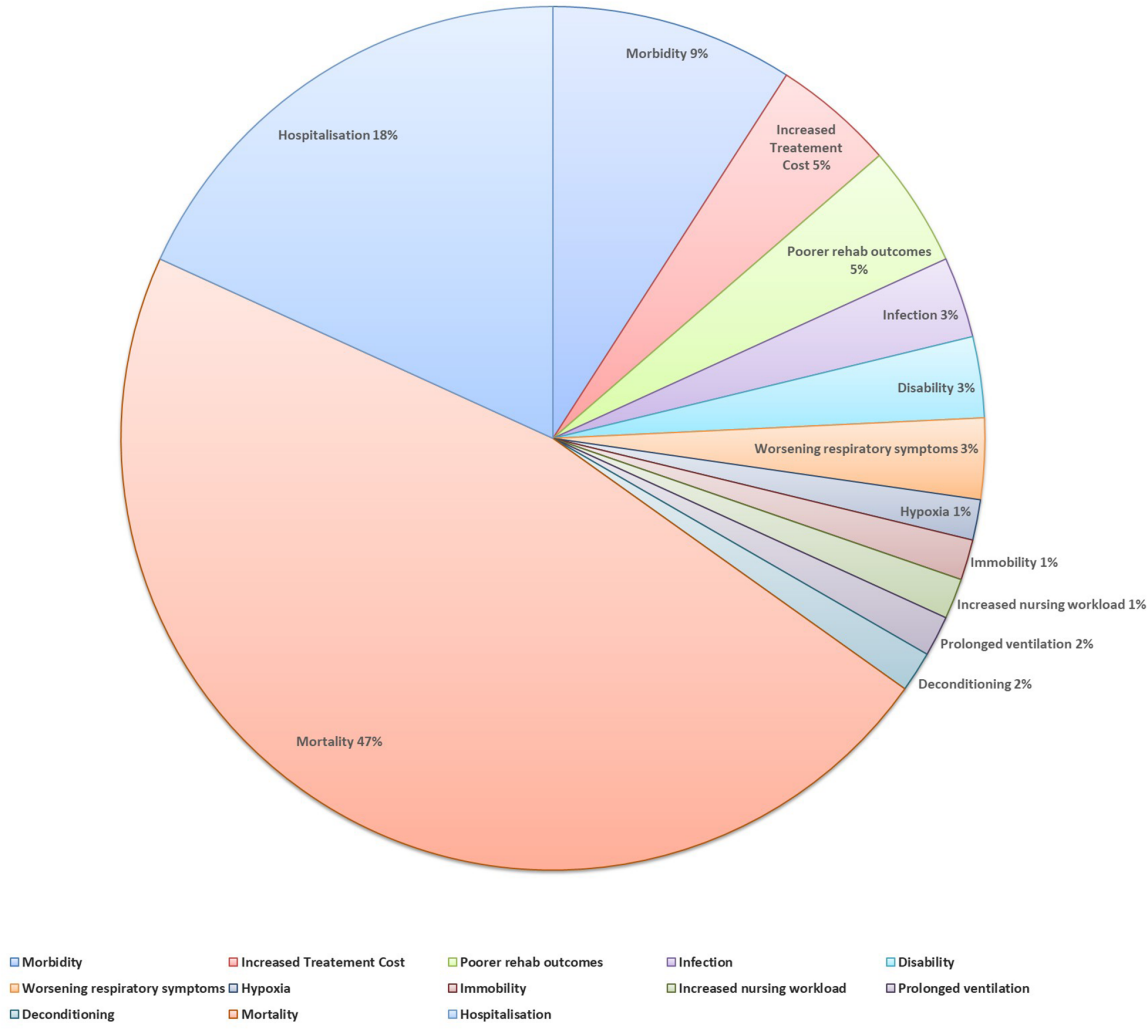
The consequences of AP.

Thirty papers (10%) described consequences of AP. Mortality was the most frequently reported (*n* = 31 papers) (8, 12, 14, 26, 36, 40, 45, 49, 53, 68, 74, 90, 102, 111, 120, 126, 137, 143, 150, 153–164). The second most common consequence was hospitalisations (*n* = 12 papers) (8, 12, 14, 34, 38, 45, 53, 95, 111, 126, 165, 166), followed by morbidity (*n* = 6 papers) (8, 45, 74, 156, 159, 161). Three papers stated AP increases treatment cost (14, 165, 166) and decreases rehabilitation outcomes (12, 14, 59). Other consequences were reported in two or less papers (Figure 7).

### 3.7 The prevention of AP

Forty-six papers (16%) described the prevention of AP. The most commonly reported way of preventing AP was appropriate management of dysphagia (*n* = 13 papers) (14, 17, 34, 36, 48, 58, 61, 63, 113, 167–170). Nurses role in preventing AP was reported in nine papers (14, 34, 36, 95, 113, 132, 167, 171, 172). In particular, five papers outline the role for nurses in screening people for dysphagia, as this can reduce the risk of AP (14, 58, 63, 113).

Another method of preventing AP was appropriate oral care, for example brushing teeth after meals, cleaning tongue, or cleaning dentures (*n* = 12 papers) (34, 39, 45, 69, 88, 90, 101, 102, 111, 173, 174). It was recommended that nurses incorporate oral care into their daily routines (88, 90). One paper reported the need for nurses to engage in periodic hands-on training to improve their knowledge and skills in providing quality oral care (90). In addition to preventing AP, oral care was noted to be potentially a cost saving option that not only improves oral health, but has a positive impact on systemic health (173).

**Figure 8:**
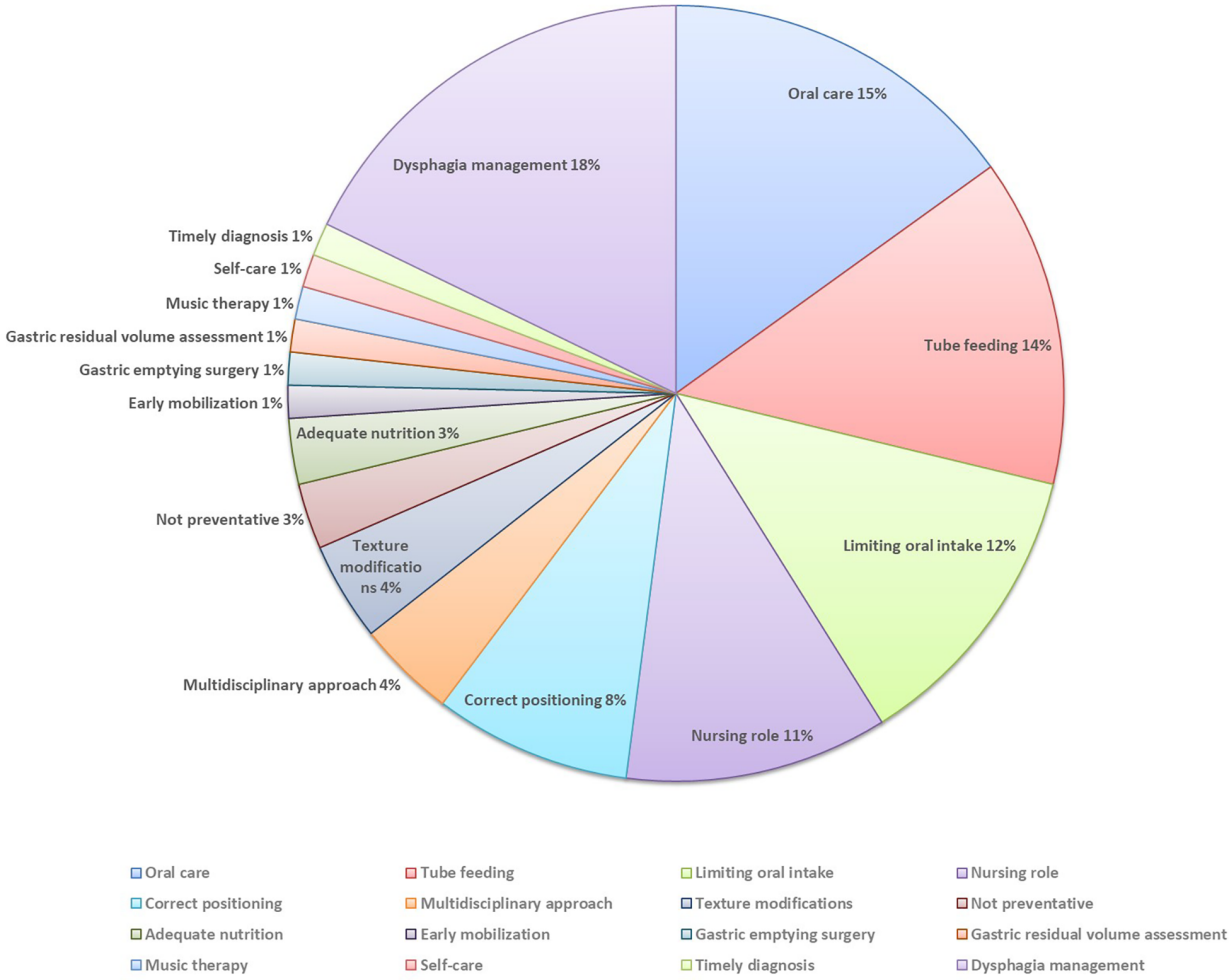
The prevention of AP.

Tube feeding was described as an option when attempting to prevent AP (*n* = 10 papers) (26, 123, 144, 157, 171, 175–179). Some papers highlighted risk factors associated with tube feeding. For example, tube feeding may increase vomiting and subsequently increase the risk of AP (26). In the same paper, the authors recommended a new nasogastric tube flushing technique, maintaining an upright body position and ensuring proper placement of feeding tube to reduce the risk of AP. It was noted that the tube feeding regime (continuous vs. intermittent) may also play a role in the prevention of AP (176). One paper drew attention to the lack of evidence to support tube feeding over oral feeding for people with advanced dementia (144). Papers also identified limiting oral intake, in the context of surgery (158, 180–183) or dysphagia (51, 113, 145, 163), as a strategy to prevent AP. Other prevention strategies are presented in the Figure 8.

### 3.8 Knowledge gaps

Five papers identified the following knowledge gaps related to AP which should be further explored:

- the mechanism between sputum suction and AP (33)
- the routine checking of gastric residuals in people receiving gastrostomy feeding and risk of AP (23)
- the evaluation of the effectiveness of the various oral health interventions in reducing AP (60, 101)
- the support for nursing education to promote oral health interventions and reduce AP (101)
- the increased demands on the nursing resources when caring for patients with poststroke complications, such as AP or nasogastric feeding (14).

## 4 Discussion

This review aimed to map how the term aspiration pneumonia has been represented in the nursing literature within the last decade. We were interested to research this literature as nursing is the largest health care profession (184) and is at the forefront of patient care delivery. This care extends to providing support to people with AP. The findings discussed below represent how the term aspiration pneumonia is perceived in nursing literature.

We discovered that 293 papers included the term aspiration pneumonia in this review. However, the volume of information regarding AP within each paper was limited. Despite this limited volume, we were able to establish that within the nursing literature, the causes, prevention, management and consequences of AP are complex, with some options, for example tube feeding, being considered as a potential help, and as a risk. We also noted that dysphagia, oral health, and tube feeding emerged across prevention, cause and management of AP. Mortality, hospitalisation and morbidity were the most frequent consequences of AP noted in this review.

Dysphagia has emerged as being closely connected to AP in this review. Dysphagia is forefront as a cause and risk factor for AP. Swallow assessment was identified as key for the management of people with/at risk of AP. In addition, the management of dysphagia is important for prevention of AP. It is recognised in the wider literature that although pharyngeal stage dysphagia may lead to aspiration (185), the relationship between aspiration and developing AP is complex and multifaceted (186, 187). There are many factors that should also be considered as potential predictors of AP particularly in older adults, including the presence of co-morbidities and individuals' mobility or ambulation (8, 188). Aspiration does not always lead to AP, for example, approximately half of healthy adults aspirate during sleep (189). Aspiration, and in particular silent aspiration, has been observed in healthy adults across the lifespan during flexible endoscopic evaluation of swallowing (190), and yet no differences have been noted between healthy older adult aspirators and non-aspirators on pulmonary computed tomography, suggesting that there may be a broad range of normal swallowing that includes asymptomatic aspiration (191).

The current review has identified that a multidisciplinary approach is perceived as important in the management of dysphagia, however the studies included in this review have not specified how exactly this approach should be provided. This multidisciplinary approach is in keeping with existing literature on the management of dysphagia across the lifespan (192–195), with one study stating that in the context of stroke an integrated team approach and pathway of care decreases the prevalence of AP (195). Dysphagia screening can be effectively performed by trained nurses and it has been shown that the early identification of dysphagia by nurses reduces AP rate in patients post stroke (196). In addition, screening undertaken by trained nurses may be effective in detecting dysphagia, reducing the time patients are kept nil-by-mouth, and is considered to be in a patient's best interest (197). Swallowing screening by a nurse may also improve the appropriateness of referrals to speech & language therapy for dysphagia services (197).

In addition to dysphagia, oral care emerged across prevention and management of AP and as a cause and risk factor for this condition. Despite the fact that oral health is considered as essential for healthy ageing, it is one of the most neglected aspects of care for older people (91, 198). Nurses play a very important role in the oral health care of their patients in terms of the assessment, planning and implementation of care. A recent scoping review has described personal care, such as oral care, to be ranked as the second fundamental of nursing in caring for older adults (199). However, research has shown that this fundamental aspect of nursing care can often be overlooked or down-prioritised (200, 201). Mitchell et al. found that most community nurses reported providing information about oral health to older adults living at home, however they were not involved in the direct provision of oral care as a routine practice and it was very much dependent on the individual patient (202). The authors called for greater interprofessional collaboration and clearer delineation of roles with oral health professionals in enhancing oral health outcomes in vulnerable groups (202).

A range of clinical cohorts may require short or long-term tube feeding (203). In our review, the link between tube feeding and AP emerged as being complex. The presence of tube feeding was identified as a risk factor for AP. Tube feeding was also identified as a management option and a mechanism of prevention of AP in the context of dysphagia. The rates of AP in people receiving tube feeding were reported between 4% and 95% in a recent review (204). AP is a common cause of death in people receiving tube feeding (204, 205). Despite the risks, tube feeding is a well-established management option for dysphagia, where nutrition and hydration requirements may not be met orally (12). The role of a nurse in supporting the safety of people receiving tube feeding has been recognised (112). This role related to nurses' involvement in swallow screening, onwards referrals, coordinating input from other professionals, and assisting the person with dysphagia during meals.

The findings of our review indicated that AP has not been researched in-depth in the nursing journals over the last decade. Our findings are sourced from journals in the field of nursing only. We have identified the complex nature of AP and the reporting of a multidisciplinary approach in the prevention and management of the condition. It is likely that this review provides only a snapshoot of literature that informs nursing practice. The research team included two nurses and two speech & language therapists therefore represented two professional perspectives within the discussion of findings.

This is the first study to glean insights into the representation of the term aspiration pneumonia in published, peer-reviewed nursing literature. However, this study has potential limitations. The eligible studies in this mapping review were not quality assessed as it was not deemed appropriate or relevant for the purpose of this study aimed at providing a descriptive overview of the published, peer reviewed nursing literature on aspiration pneumonia. The search undertaken for this study was limited to one Higher Education Institution library and refined to peer-reviewed journals. Records such as book chapters were excluded which may have limited insights into the topic. However, due to the volume of potential literature we decided that focusing on peer-reviewed journal articles was the preferred option for logistical reasons.

In conclusion, this mapping review highlights the lack of attention given to the topic of AP in the nursing literature, as evidenced by the overall limited focus on AP within the eligible studies reviewed. Despite this, the studies reviewed found a diverse range of causes or risk factors for developing AP, with dysphagia, poor oral health and hygiene, and tube feeding identified as the most common. The study reveals that the management of AP requires a multidisciplinary approach and nurses play a central role with responsibility for a complex range of care interventions, including dysphagia assessment, diet modifications, tube feeding and oral care. Many consequences of AP were identified in the review, such as mortality, hospitalisation and morbidity. Preventive strategies for AP in terms of appropriate management of dysphagia, oral care and tube feeding were illustrated. The study highlights a number of knowledge gaps for future research. Further research on the nurses' role in the management of AP and dysphagia, as well as their involvement in providing oral care, is indicated. In addition, country and culturally specific differences in the management of AP should be explored.

## Data Availability

The original contributions presented in the study are included in the article/Supplementary Material, further inquiries can be directed to the corresponding author.
