# Supplementary material for: Aspiration pneumonia in nursing literature—a mapping review
Source: Front Rehabil Sci. 2024 Jul 24;5:1393368. doi: 10.3389/fresc.2024.1393368 (PMC11304538; doi:10.3389/fresc.2024.1393368)
Supplement: Supplementary file 1 [file Table1.docx]

Supplementary Material

| **Database** | **Journal title** | **No of records** |
| --- | --- | --- |
| **Science Direct** | 1. Advances in family practice nursing | 1 |
|  | 1. Applied nursing research | 6 |
|  | 1. Archives of psychiatric nursing | 0 |
|  | 1. Clinical effectiveness in nursing | 0 |
|  | 1. Clinical simulation in nursing | 3 |
|  | 1. Collegian (Royal College of Nursing, Australia) | 5 |
|  | 1. European journal of oncology nursing: the official journal of European Oncology Nursing Society | 3 |
|  | 1. Geriatric nursing (New York) | 22 |
|  | 1. Intensive & critical care nursing | 10 |
|  | 1. International emergency nursing | 5 |
|  | 1. International journal of nursing studies | 19 |
|  | 1. International journal of orthopaedic and trauma nursing | 0 |
|  | 1. Journal of emergency nursing | 6 |
|  | 1. Journal of neonatal nursing : JNN | 2 |
|  | 1. Journal of nursing regulation | 0 |
|  | 1. Journal of pediatric nursing | 3 |
|  | 1. Journal of perianesthesia nursing | 15 |
|  | 1. Journal of professional nursing | 1 |
|  | 1. Journal of radiology nursing | 5 |
|  | 1. Journal of vascular nursing | 3 |
|  | 1. Newborn and infant nursing reviews | 1 |
|  | 1. Nurse education in practice | 5 |
|  | 1. Nurse education today | 3 |
|  | 1. Nurse leader | 1 |
|  | 1. Nursing outlook | 1 |
|  | 1. Pain management nursing | 1 |
|  | 1. Seminars in oncology nursing | 4 |
|  | 1. Teaching and learning in nursing | 0 |
|  |  | **N = 125 papers** |
| **CINAHL Complete** | 1. Advances in nursing science | 0 |
|  | 1. Africa journal of nursing and midwifery. | 0 |
|  | 1. American nurse today | 5 |
|  | 1. BMC nursing (also academic search complete & health research premium collection) | 13 |
|  | 1. British journal of cardiac nursing | 0 |
|  | 1. British journal of community nursing | 5 |
|  | 1. British journal of neuroscience nursing | 16 |
|  | 1. British journal of nursing (Mark Allen Publishing) | 39 |
|  | 1. British Journal of Primary Care Nursing. Cardiovascular Disease, Diabetes, Stroke, Kidney & Liver Care | 0 |
|  | 1. Canadian journal of cardiovascular nursing | 1 |
|  | 1. Canadian journal of emergency nursing | 0 |
|  | 1. Canadian journal of neuroscience nursing | 0 |
|  | 1. Canadian Journal of Neuroscience Nursing / Journal Canadien des Infirmières et Infirmiers en Neurosciences | 0 |
|  | 1. Canadian oncology nursing journal | 0 |
|  | 1. Central European Journal of Nursing and Midwifery | 0 |
|  | 1. Clinical journal of oncology nursing | 2 |
|  | 1. Comprehensive child and adolescent nursing | 0 |
|  | 1. Critical care nurse | 29 |
|  | 1. Critical care nursing quarterly | 0 |
|  | 1. Dermatological nursing | 0 |
|  | 1. Dermatology nursing | 0 |
|  | 1. Dimensions of critical care nursing | 1 |
|  | 1. Gastrointestinal nursing | 4 |
|  | 1. Holistic nursing practice | 0 |
|  | 1. International Journal of Community Based Nursing & Midwifery | 1 |
|  | 1. International journal of palliative nursing | 1 |
|  | 1. The international journal of psychiatric nursing research | 0 |
|  | 1. ISRN nursing | 0 |
|  | 1. JARNA : the official journal of the Australasian Rehabilitation Nurses' Association | 0 |
|  | 1. Journal of aesthetic nursing | 0 |
|  | 1. The Journal of cardiovascular nursing | 0 |
|  | 1. Journal of children's and young people's nursing | 0 |
|  | 1. Journal of Diabetic Nursing | 0 |
|  | 1. Journal of hospice and palliative nursing | 0 |
|  | 1. Journal of Korean Academy of Nursing | 3 |
|  | 1. Journal of Korean Academy of Nursing Administration | 1 |
|  | 1. The journal of multicultural nursing & health | 0 |
|  | 1. Journal of Nursing & Midwifery Sciences | 0 |
|  | 1. Journal of nursing and healthcare of chronic illness | 0 |
|  | 1. Journal of nursing care quality | 1 |
|  | 1. Journal of nursing law | 0 |
|  | 1. Journal of nursing practice applications & reviews of research | 0 |
|  | 1. The journal of nursing research | 0 |
|  | 1. The Journal of perinatal & neonatal nursing | 0 |
|  | 1. Journal of perioperative nursing | 0 |
|  | 1. The Journal of the New York State Nurses Association | 0 |
|  | 1. Journal of trauma nursing | 2 |
|  | 1. The Kansas nurse | 0 |
|  | 1. Kibon Kanho Hakhoe chi | 0 |
|  | 1. Medsurg nursing | 13 |
|  | 1. Mental health nursing (London, England) | 0 |
|  | 1. Neonatal, paediatric, and child health nursing | 0 |
|  | 1. Norwegian Journal of Clinical Nursing / Sykepleien Forskning | 0 |
|  | 1. Nuritinga electronic journal of nursing | 0 |
|  | 1. The Nurse practitioner | 0 |
|  | 1. Nursing administration quarterly | 0 |
|  | 1. Nursing & residential care | 11 |
|  | 1. Nursing economic$ | 0 |
|  | 1. Nursing leadership (Toronto, Ont.) | 0 |
|  | 1. Nursing management | 0 |
|  | 1. Nursing New Zealand (Wellington, N.Z. : 1995) | 0 |
|  | 1. Nursing practice today | 2 |
|  | 1. Nursing reports (Pavia, Italy) | 3 |
|  | 1. Nursing: Theory, Research, Education / Osetrovatel'stvo: Teória, Výskum, Vzdelávanie | 0 |
|  | 1. Oncology nursing forum | 6 |
|  | 1. Online Brazilian journal of nursing | 0 |
|  | 1. Online journal of issues in nursing | 1 |
|  | 1. Online journal of nursing informatics | 0 |
|  | 1. Online journal of rural nursing and health care | 0 |
|  | 1. Pacific Rim international journal of nursing research | 0 |
|  | 1. Pediatric nursing | 0 |
|  | 1. Perspectives: The Journal of the Canadian Gerontological nursing Association | 0 |
|  | 1. Polish Nursing / Pielegniarstwo Polskie | 0 |
|  | 1. Practice nursing | 0 |
|  | 1. Professional nursing today Online | 0 |
|  | 1. Progress in cardiovascular nursing | 0 |
|  | 1. Tennessee nurse | 0 |
|  | 1. West African journal of nursing | 0 |
|  |  | **N = 160 papers** |
| **JSTOR life sciences** | 1. The American journal of nursing | 0 |
| **Health Research Premium Collection** | 1. Asian nursing research | 1 |
|  | 1. Australian journal of advanced nursing | 0 |
|  | 1. British journal of anaesthetic and recovery nursing | 0 |
|  | 1. Canadian journal of nursing informatics | 0 |
|  | 1. Canadian nurse (1924)(also CINAHL) | 0 |
|  | 1. Cancer nursing practice | 0 |
|  | 1. Contemporary nurse : a journal for the Australian nursing profession | 0 |
|  | 1. Creative nursing | 1 |
|  | 1. Emergency nurse | 6 |
|  | 1. International journal of nursing education scholarship | 0 |
|  | 1. JMIR nursing | 0 |
|  | 1. Journal for nurse practitioners | 12 |
|  | 1. The Journal of continuing education in nursing | 7 |
|  | 1. Journal of doctoral nursing practice | 1 |
|  | 1. Journal of gerontological nursing | 7 |
|  | 1. The Journal of neuroscience nursing | 0 |
|  | 1. Journal of Nursing and Midwifery Sciences | 0 |
|  | 1. The Journal of nursing education | 4 |
|  | 1. Journal of nursing measurement | 1 |
|  | 1. Journal of nursing scholarship | 3 |
|  | 1. The Journal of practical nursing |  |
|  | 1. Journal of psychosocial nursing and mental health services | 0 |
|  | 1. Mental Health Nursing (Online) | 0 |
|  | 1. Nephrology nursing journal : journal of the American Nephrology Nurses' Association | 0 |
|  | 1. Nordic journal of nursing research | 2 |
|  | 1. Nurse prescriber | 0 |
|  | 1. Nurse researcher | 0 |
|  | 1. Nursing children and young people | 4 |
|  | 1. Nursing education perspectives | 3 |
|  | 1. Nursing history review | 0 |
|  | 1. Nursing journal of India | 0 |
|  | 1. Nursing leadership forum | 0 |
|  | 1. Nursing management (Harrow, London, England) | 0 |
|  | 1. Nursing older people | 8 |
|  | 1. Nursing praxis in New Zealand inc. | 1 |
|  | 1. Nursing : research and reviews (Auckland, N.Z.) | 3 |
|  | 1. Nursing standard | 29 |
|  | 1. ONS nursing scan in oncology | 0 |
|  | 1. Orthopaedic nursing | 0 |
|  | 1. Perspectives - Gerontological Nursing Association | 0 |
|  | 1. Research and theory for nursing practice | 0 |
|  | 1. Research in gerontological nursing | 6 |
|  | 1. Urologic nursing | 0 |
|  |  | **N = 99 papers** |
| **SAGE Journals Premier 2021 (PREM2021)** | 1. Biological research for nursing | 1 |
|  | 1. Canadian journal of nursing research | 2 |
|  | 1. Clinical nursing research | 5 |
|  | 1. Journal of family nursing | 5 |
|  | 1. Journal of holistic nursing | 0 |
|  | 1. Journal of pediatric hematology/oncology nursing | 1 |
|  | 1. Journal of research in nursing | 2 |
|  | 1. The Journal of school nursing | 1 |
|  | 1. Journal of the American Psychiatric Nurses Association | 1 |
|  | 1. Journal of transcultural nursing | 1 |
|  | 1. NASN school nurse | 1 |
|  | 1. Nursing ethics | 6 |
|  | 1. Nursing science quarterly | 1 |
|  | 1. Policy, politics & nursing practice | 1 |
|  | 1. SAGE open nursing | 6 |
|  | 1. Western journal of nursing research | 2 |
|  |  | **N = 36 papers** |
| [**SAGE Journals GOLD OA 2023**](https://rq9bu4lb4w.search.serialssolutions.com/log?L=RQ9BU4LB4W&D=AFCOW&J=TC0001340586&P=EJP&PT=EZProxy&A=Global+qualitative+nursing+research&H=0a58565e58&U=https%3A%2F%2Fmtu.idm.oclc.org%2Flogin%3Furl%3Dhttps%3A%2F%2Fjournals.sagepub.com%2Floi%2Fgqn%3Futm_source%3Dss360%26utm_medium%3Ddiscovery-provider) | 1. Global qualitative nursing research | 2 |
|  |  | **N = 2 papers** |
| [**Oxford Journals 2023 All Titles**](https://rq9bu4lb4w.search.serialssolutions.com/log?L=RQ9BU4LB4W&D=AAPXW&J=EUROJOUROFCAN&P=EJP&PT=EZProxy&A=European+journal+of+cardiovascular+nursing+%3A+journal+of+the+Working+Group+on+Cardiovascular+Nursing+of+the+European+Society+of+Cardiology&H=cb4f658cdb&U=https%3A%2F%2Fmtu.idm.oclc.org%2Flogin%3Furl%3Dhttps%3A%2F%2Facademic.oup.com%2Feurjcn) | 1. European journal of cardiovascular nursing : journal of the Working Group on Cardiovascular Nursing of the European Society of Cardiology | 9 |
|  |  | **N = 9 papers** |
| **Wiley Online Library All Journals** | 1. International journal of mental health nursing | 3 (abstracts from conference) |
|  | 1. International journal of nursing knowledge | 8 |
|  | 1. International journal of nursing practice | 9 |
|  | 1. International journal of older people nursing | 6 |
|  | 1. International journal of urological nursing | 0 |
|  | 1. International nursing review | 3 |
|  | 1. Japan journal of nursing science : JJNS | 11 |
|  | 1. Journal for specialists in pediatric nursing | 5 |
|  | 1. Journal of advanced nursing | 14 |
|  | 1. Journal of child and adolescent psychiatric nursing | 0 |
|  | 1. Journal of clinical nursing | 56 |
|  | 1. Journal of forensic nursing | 0 |
|  | 1. Journal of nursing management | 7 |
|  | 1. Journal of obstetric, gynecologic, and neonatal nursing | 5 |
|  | 1. Journal of psychiatric and mental health nursing | 2 |
|  | 1. Journal of the American Association of Nurse Practitioners | 12 |
|  | 1. Nursing & health sciences | 2 |
|  | 1. Nursing for women's health | 0 |
|  | 1. Nursing forum (Hillsdale) | 1 |
|  | 1. Nursing in critical care | 7 |
|  | 1. Nursing open | 15 |
|  | 1. Nursing philosophy | 0 |
|  | 1. Public health nursing (Boston, Mass.) | 0 |
|  | 1. Rehabilitation nursing | 2 |
|  | 1. Research in nursing & health | 1 |
|  | 1. Worldviews on evidence-based nursing | 4 |
|  |  | **N = 173 papers** |
| **Academic search complete** | 1. Iranian journal of nursing and midwifery research | 0 |
|  | 1. Journal of psychiatric nursing (Istanbul, Turkey) | 0 |
|  | 1. Nursing (Jenkintown, Pa.) | 0 |
|  | 1. Nursing Research and Practice | 0 |
|  |  | **N = 0 papers** |
| [**OA Read&Publish Medical 2022**](https://rq9bu4lb4w.search.serialssolutions.com/log?L=RQ9BU4LB4W&D=AGFJD&J=ISSUINMENHE&P=EJP&PT=EZProxy&A=Issues+in+mental+health+nursing&H=d5cd76c235&U=https%3A%2F%2Fmtu.idm.oclc.org%2Flogin%3Furl%3Dhttps%3A%2F%2Fwww.tandfonline.com%2Floi%2Fimhn20) | 1. Issues in mental health nursing | 0 |
|  | 1. Journal of community health nursing | 1 |
|  |  | **N = 1 paper** |
|  |  | **Total: n = 605** |
